# Supplementary material for: Patterns of Intron Gain and Loss in Fungi
Source: PLoS Biol. 2004 Nov 30;2(12):e422. doi: 10.1371/journal.pbio.0020422 (PMC532390; doi:10.1371/journal.pbio.0020422)
Supplement: Table S1 — Also available at http://genes.mit.edu/NielsenEtAl/. (4.3 MB ZIP). [file pbio.0020422.st001.zip › NielsenEtAl/html/776.html]

AN2738.1.NCU08607.1.MG01245.1.FG00369.1


```
 CLUSTAL W (1.82) Multiple Sequence Alignments - Introns Inserted


Sequence 1: NCU08607.1	444 aa
Sequence 2: MG01245.1	444 aa
Sequence 3: FG00369.1	444 aa
Sequence 4: AN2738.1	437 aa
Alignment Length: 459 aa
Number Identitical Residues: 248 aa
Alignment Score (without introns) 12227


MG01245.1 	MAPKSRFTRLDAFTKTVEDARIRTTSGGIITIVSLIVVLYLAWGEWADYRRIDIHPELIV
NCU08607.1	MAGKWRFTKLDAFTKTVEDARIRTTSGGIVTIVSLLVVLFLSWGEWRDYRKVVIHPELVV
FG00369.1 	MPPKSRFTRLDAFTKTVDEARIRTTSGGVVTIVSLLVVLFLSWGEWADYRRIDIHPELIV
AN2738.1  	MAAKSRFTRLDAFAKTVDEARIRTTSGGIITIASLLIIIWLTWGEWVDYRRVAVLPELVV
          	*. * ***:****:***::*********::**.**:::::*:**** ***:: : ***:*

MG01245.1 	DKSRG1DRMEIHLNITFPRVPCELLTLDVMDVSGEQQHGVQHGVIKVRLRPQSEGGGVID
NCU08607.1	DKGRG1ERMEIHLNITFPKVPCELLTLDVMDVSGEQQHGVQHGVKKIRLRPQSEGGGEID
FG00369.1 	DKGRG1ERMEIHLNITFPKMPCELLSLDVMDVSGEQQHGVMHGVNKVRLQPESQGGAVID
AN2738.1  	DKSRG~EKMEIHLNITFPRLPCELTTLDVMDVSGEQQVGVAHGVNKVRLAPAAEGGRVLD
          	**.** ::**********::**** :*********** ** *** *:** * ::**  :*

MG01245.1 	AKTLAL~HAEDEAATHLDPNYCGGCYGAPAPANAKKAGCCNTCDEVREAYAQASWAFGRG
NCU08607.1	AKVLSL2HAADESATHLDPSYCGPCYGAPAPYNAKKPGCCSTCEEVREAYAQASWAFGDG
FG00369.1 	TKSLSL~H--DDAAHHLDPSYCGGCYGATPPANAQKAGCCQTCDEVREAYAQASWAFGRG
AN2738.1  	VQALQL2H--AEEAKHLDPDYCGECGGAPPPPNAIKPGCCSTCDEVREAYAQKQWGFGKG
          	.: * * *   : * ****.*** * **..* ** *.***.**:******** .*.** *

MG01245.1 	ENVEQCTREHYAERLDEQRHEGCQIAGSLRVNKVVGNFHLAPGRSFSNGNMHVHDLKNYW
NCU08607.1	ATMEQCQREHYTERLAEQRHEGCRIEGGLRVNKVIGNFHIAPGRSFSNGNMHVHDLAQWW
FG00369.1 	EGVEQCEREHYGEKLDAQRSEGCRIEGGLRVNKVIGNFHFAPGRSFSSGNMHVHDLKNYW
AN2738.1  	TNIEQCEREHYSERIDAQRREGCRLEGVIRVNKVVGNFHIAPGRSFSSNNVHIHDIANYE
          	  :*** **** *::  ** ***:: * :*****:****:*******..*:*:**: :: 

MG01245.1 	D--TPVEGGHSFSHTIHSLRFGPQLPPSALEKLGNKDKNMPWTN-HHINPLDGVIQTTVD
NCU08607.1	S--TPVPGGHSFSHIIHSLRFGPQLPDDLVRKLGGNGKNTLWTN-HHLNPLDNTKQETND
FG00369.1 	D--VPKGFSHDFTHIVHSLRFGPQLPDHIARKVG--HKNTLWTN-HHQNPLDDTRQETHD
AN2738.1  	ERGLSPAEQHTMSHIIHSLRFGPQLPDELSDRWQ-------WTDHHHTNPLDSTSQEAPE
          	. . .    * ::* :**********     :         **: ** ****.. * : :

MG01245.1 	PNFNYMYFVKIVPTSYLPLGWEKR---THLATMHDH--GVGTYGYSG--DGSVETHQYSV
NCU08607.1	PNYNFMYFVKIVPTSYLPLGWEKQAAQNKAAWEQDHSVGLGAYGYGS--DGSMETHQYSV
FG00369.1 	PNYNFMYFVKIVPTSYLPLGWDKKG-IKIAGLLQEDNAGLGAYGYGE--DGSVETHQYSV
AN2738.1  	PAYSFMYFIKVVSTSYLPLGWDPLY-SASLHAAADTNTPLGAQGLSAGSQGSIETHQYSV
          	* :.:***:*:*.********:            : .  :*: * . .::**:*******

MG01245.1 	TSHKRSLAGGDDGEDGHKERMHSRGGIPGVFFSY0PFCPQDISPMKVINREVRTKTFAGF
NCU08607.1	TSHKRSLTGGDDSKEGHGERLHSRGGIPGVFFSY0-----DISPMKVVNREERAKSFLGF
FG00369.1 	TSHRRSLAGGNDAAEGHAERQHTSGGIPGVFFSY0-----DISPMKVVNREEKAKTFSGF
AN2738.1  	TSHKRSLRGGDASDEAHKERIHAAGGIPGVFFNY~-----DISPMKVINREARPKTFTGF
          	***:*** **: . :.* ** *: ********.*      *******:*** :.*:* **

MG01245.1 	LTGLCAILGGTLTVAAAIDRMTFEGVTRIKKMQSKNL~-----
NCU08607.1	LAGLCAVVGGTLTVAAAVDRGLFEGTVRLKKLRSKDN~-----
FG00369.1 	LAGLCAIVGGTLTVAAAVDRGLFEGAARLKKMRSKDM2VDVLD
AN2738.1  	LTGVCAIVGGTLTVAAAIDRTLYEGVSRVRKLHSN--~-----
          	*:*:**::*********:**  :**. *::*::*:
```
